# TRAIL-receptor preferences in pancreatic cancer cells revisited: Both TRAIL-R1 and TRAIL-R2 have a licence to kill

**DOI:** 10.1186/s12885-015-1508-2

**Published:** 2015-07-03

**Authors:** Andrea Mohr, Rui Yu, Ralf M. Zwacka

**Affiliations:** 1 School of Biological Sciences, University of Essex, Wivenhoe Park, Colchester, CO4 3SQ United Kingdom; 2 School of Medicine, Ningbo University, Ningbo, Zhejiang 315211 P.R. China

**Keywords:** TRAIL, Pancreatic cancer, DR4 specific TRAIL variant, DR5 specific TRAIL variant, Apoptosis, TRAIL receptor

## Abstract

**Background:**

TRAIL is a potent and specific inducer of apoptosis in tumour cells and therefore is a possible new cancer treatment. It triggers apoptosis by binding to its cognate, death-inducing receptors, TRAIL-R1 and TRAIL-R2. In order to increase its activity, receptor-specific ligands and agonistic antibodies have been developed and some cancer types, including pancreatic cancer, have been reported to respond preferentially to TRAIL-R1 triggering. The aim of the present study was to examine an array of TRAIL-receptor specific variants on a number of pancreatic cancer cells and test the generality of the concept of TRAIL-R1 preference in these cells.

**Methods:**

TRAIL-R1 and TRAIL-R2 specific sTRAIL variants were designed and tested on a number of pancreatic cancer cells for their TRAIL-receptor preference. These sTRAIL variants were produced in HEK293 cells and were secreted into the medium. After having measured and normalised the different sTRAIL variant concentrations, they were applied to pancreatic and control cancer cells. Twenty-four hours later apoptosis was measured by DNA hypodiploidy assays. Furthermore, the specificities of the sTRAIL variants were validated in HCT116 cells that were silenced either for TRAIL-R1 or TRAIL-R2.

**Results:**

Our results show that some pancreatic cancer cells use TRAIL-R1 to induce cell death, whereas other pancreatic carcinoma cells such as AsPC-1 and BxPC-3 cells trigger apoptosis via TRAIL-R2. This observation extended to cells that were naturally TRAIL-resistant and had to be sensitised by silencing of XIAP (Panc1 cells). The measurement of TRAIL-receptor expression by FACS revealed no correlation between receptor preferences and the relative levels of TRAIL-R1 and TRAIL-R2 on the cellular surface.

**Conclusions:**

These results demonstrate that TRAIL-receptor preferences in pancreatic cancer cells are variable and that predictions according to cancer type are difficult and that determining factors to inform the optimal TRAIL-based treatments still have to be identified.

## Background

Pancreatic cancers are one of the most serious oncological diseases, for which novel treatment options are urgently needed. TRAIL is a cytokine that is involved in natural tumour surveillance mechanisms and as recombinant protein has been shown to exert specific anti-tumour effects by induction of apoptosis in cancer cells [1–5]. Apoptosis is triggered after binding of TRAIL to one of its two receptors, TRAIL-receptor 1 (TRAIL-R1) or TRAIL-receptor 2 (TRAIL-R2), also known as DR4 and DR5, respectively [6–8]. Binding of TRAIL to these two receptors stimulates the formation of a protein complex called the death-inducing signaling complex (DISC). It consists of TRAIL-R1 and/or TRAIL-R2, the adaptor protein Fas-associated death domain (FADD) and procaspase-8. At the DISC, caspase-8 is activated by a mechanism that involves dimerisation and proteolytic cleavage [9, 10]. Active caspase-8 can then, either directly, or indirectly via the BH3-only protein Bid, activate effector caspases, such as caspase-3, which in turn cleave many cellular substrates resulting in the biochemical and morphological features characteristic of apoptosis [11]. Aside from the two death domain (DD)-containing, apoptosis-inducing receptors, TRAIL-R1 and TRAIL-R2, three additional decoy receptors exist, TRAIL-R3 (DcR1), TRAIL-R4 (DcR2) and Osteoprogerin (OPG) [6, 7, 12–14]. These decoy receptors can inhibit the apoptosis-inducing function of TRAIL [15]. To address this issue, agonistic antibodies against either TRAIL-R1 or TRAIL-R2 have been developed and have been tested in pre-clinical and as well as clinical studies [16–21].

In addition, engineered variants of TRAIL, containing specific amino acid changes leading to specific targeting of TRAIL-R1 or TRAIL-R2 have been designed and have shown improved anti-tumour effects *in-vitro* and *in-vivo* when compared to wild-type TRAIL [22–27]. Such TRAIL-receptor variants have been studied in the context of various specific cancer types as well as in the context of combination treatments [28–32]. TRAIL variants might hold important advantages over TRAIL-receptor specific antibodies as they are smaller than antibodies and might therefore be better able to reach and infiltrate growing tumours. In addition, such proteins can be further optimised to increase activity, specificity and stability and they can be used as part of gene and cell therapeutic approaches [31, 33–38]. This way of potentially improving the therapeutic efficacy of TRAIL by using TRAIL-receptor specific agents is of particular interest for pancreatic cancer, as previous studies have shown that pancreatic tumour cells preferentially use TRAIL-R1 to execute TRAIL-induced apoptosis [39, 40]. Thus, agonistic TRAIL-R1 specific antibodies or TRAIL-R1 targeting variants of TRAIL were regarded as having a higher therapeutic potential than normal TRAIL in the treatment of pancreatic carcinoma.

We wondered, given the molecular heterogeneity of tumours, how such a uniform TRAIL response with respect to receptor preferences could be possible. Therefore, we set out to examine an array of pancreatic cancer cells for their TRAIL-receptor preferences. We found that a number of pancreatic cancer cells used TRAIL-R2 rather than TRAIL-R1 to initiate apoptosis signalling. These results demonstrate that, while TRAIL-receptor specific variants constitute a potentially substantial improvement to conventional TRAIL therapies, generalised predictions according to cancer type are difficult. Therefore, additional research is needed to identify factors that determine the optimal TRAIL variant (or antibody) on a case-by-case basis for each individual tumour.

## Methods

### Reagents and cell culture

All reagents were purchased from Sigma (St. Louis, MO) unless otherwise stated. The human pancreatic cancer cell lines Panc1 and PancTu1, the human embryonic kidney cell line HEK293, the human colon cancer cell line Colo205 and the human cervix carcinoma cell line HeLa were maintained in Dulbecco’s modified Eagle’s medium (DMEM). The human pancreatic cancer cell lines AsPC-1, BxPC-3 and Colo357 were cultured in RPMI-1640 medium. The human colorectal cancer cell line HCT116 was cultured in McCoy’s medium and the human prostate cancer PC-3 cells were grown in Ham’s F12 medium. All media were supplemented with 10 % FBS, 100 U/ml penicillin and 100 μg/ml streptomycin. Cells were cultured in a humidified incubator at 37 °C and 5 % CO_2_.

### Generation of sTRAIL constructs

Generation of sTRAIL constructs and site-directed mutagenesis have been previously described [31]. Briefly, the soluble portion of human TRAIL (amino acids 114–281) was first subcloned into the NheI/NotI sites of a pcDNA3 plasmid (Invitrogen) giving rise to pcDNA3.sTRAIL. Then an exogenous signal peptide sequence of the human fibrillin protein, the Furin cleavage site (Furin CS) and Isoleucine-zipper sequence (ILZ) cassette was cloned into the BamHI/NheI sites of the pcDNA3.sTRAIL vector. The resulting vector was termed sTRAIL^wt^. The two sTRAIL^DR5^ and three sTRAIL^DR4^ constructs were generated using the Quick-Change site-directed mutagenesis kit (Stratagene, La Jolla, CA) and confirmed by DNA sequencing.

### TRAIL Enzyme-linked Immunosorbent Assay (ELISA)

TRAIL concentrations were measured by a human TRAIL/TNFSF10 Quantikine ELISA Kit as recommended by the manufacturer (R&D Systems, Minneapolis, MN). Before the measurement the medium supernatants were pipetted off the respective HEK293 producer cells and then centrifuged to clear them of any cellular debris.

### TRAIL receptor surface stain

For the TRAIL receptor stain we used monoclonal anti-TRAIL-R1 (DJR1) and anti-TRAIL-R2 (DJR2-4) antibodies (1 μg/10^6^ cells; BioLegend, San Diego, CA) that were conjugated to Phycoerythrin (PE). The isotype control antibody (MOPC-21) (1 μg/10^6^ cells) was also purchased from BioLegend. The surface expression of TRAIL receptors was measured by incubating cells with the PE-conjugated mouse anti-human TRAIL-R1 and mouse anti-human TRAIL-R2 antibodies as described previously [41].

### Transfection of HEK293 cells

HEK293 cells were transfected using the Calcium-phosphate method. Briefly, before transfection, fresh 2 % FBS containing medium was added to the cells. For each well of a 6-well plate, 0.5 ml HBS were aliquoted into a sterile 1.5 ml Eppendorf tube. In a separate tube 5 μg of plasmid DNA were mixed with 250 μl CaCl_2_ (2.5 mM) and sterile water added to 0.5 ml. The CaCl_2_/DNA mix was then added to the HBS in a drop-wise fashion and constant vortexing at slow speed. After 45 min of incubation at room temperature, the mixture was slowly added to the cells. After 4 h, the medium was removed and the cells were washed with PBS and fresh growth medium added.

### Apoptosis assay

Apoptosis was measured according to Nicoletti et al. (DNA hypoploidy assay) and has been described previously [42, 43]. Trypsinised cells including the supernatant medium and PBS wash-solution were directly transferred into FACS tubes and centrifuged at 1,300 rpm for 7 min at 4 °C. After washing the cell pellet with PBS, Nicoletti buffer (Sodium citrate 0.1 % (w/v) supplemented with 0.1 % Triton X-100 (w/v) and propidium iodide at 50 μg/ml) was added. Then the tubes were vortexed for 10 s at medium speed and left for 5 h in a refrigerator. The fluorescence intensity was then measured by flow cytometry and analysed using the Venturi One software package (Applied Cytometry, Sheffield, UK). Where specified, untreated cells were taken as reference to calculate specific apoptosis by subtraction of the basal cell death values from the apoptosis levels of treated cells.

### RNAi knock-down constructs and stable cell line generation

The following small hairpin (sh) RNA motifs were used to silence: DR5 (5′-GCTAGAAGGTAATGCAGACTCTGCCATGTC -3’), DR4 (5′-GCTGTTCTTTGACAAGTTGC-3’) and XIAP (5′-GTGGTAGTCCTGTTTCAGC-3’). Sense and antisense oligos containing the sh-sequence and a 5’ overhang representing a restricted BbsI site and EcoRI site on the 3’ side were hybridised to generate double-stranded DNA fragments. These fragments were then cloned into a BbsI/EcoRI opened up pU6.ENTR plasmid (Life Technologies, Carlsbad, CA). The resulting pU6.ENTR plasmids (pU6.ENTR.shDR5, pU6.ENTR.shDR4, pU6.ENTR.shXIAP) were used to generate the pBlock-iT.shDR5, pBlock-iT.shDR4 and pBlock-iT.shXIAP plasmids using the pBLOCK-iT6-DEST vector (Life Technologies) and LR Clonase II. This was used to generate stable DR5 and DR4 knock-down clones of HCT116 cells and stable XIAP knock-down clones of PancTu1 and Panc1 cells. For this, the pBlock-iT.shDR5, pBlock-iT.shDR4 and pBlock-iT.shXIAP plasmids were FuGeneHD-transfected (Roche, Basle, Switzerland) into HCT116, PancTu1 and Panc1 cells, respectively. Three days later, the transfected cells were split into Blasticidin containing selection medium. Clones were then picked, transferred to 24 well-plates and analysed for DR5, DR4 and XIAP knock-down, respectively. Clones that did not show a knock-down were used as controls and labelled PancTu1.shctrl and Panc1.shctrl, respectively. These control clones were tested and shown to behave like parental cells.

### Statistical analysis

Experimental values are expressed as mean value ± standard error (SEM). For significance analyses, analysis of variance (ANOVA) between groups was used and *P* < 0.05 (*) was considered significant.

## Results

### Expression and specificity of DR4- and DR5-specific TRAIL variants

**Fig. 1:**
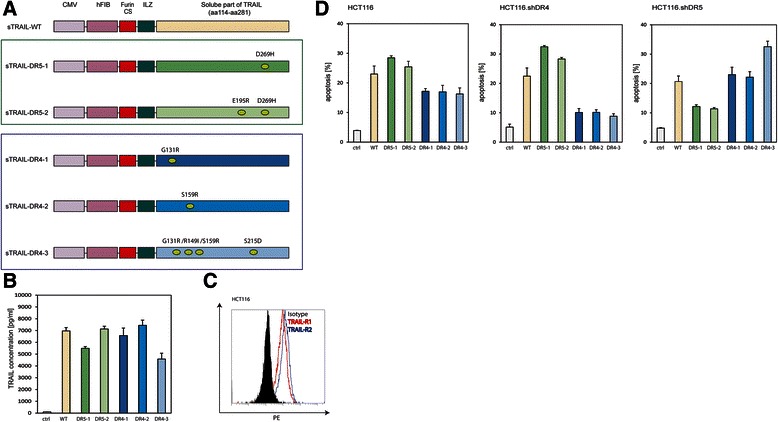
Design, expression and specificity of sTRAIL specific variants. **a** Schematic drawing of sTRAIL constructs, all of which contain a heterologous signal peptide sequence from the human fibrillin-1 gene (hFIB) ligated to a Furin cleavage site (Furin CS), an Isoleucine Zipper (ILZ) domain and the soluble part of TRAIL (aa114-aa281). The expression was driven by a conventional CMV promoter/enhancer element (CMV). The mutations in sTRAIL^wt^ leading to the two sTRAIL^DR5^ (TRAIL-R2 specific) and three sTRAIL^DR4^ (TRAIL-R1 specific) variants are shown in the respective sTRAIL segments. **b** Results of ELISA analyses for TRAIL showing the levels of secreted sTRAIL^wt^ (yellow), sTRAIL^DR5–1^ (dark green), sTRAIL^DR5–2^ (light green), sTRAIL^DR4–1^ (dark blue), sTRAIL^DR4–2^ (light blue) and sTRAIL^DR4–3^ (blue-grey) into the supernatant of HEK293 cells that were transfected with the described constructs. Results for cells transfected with an EGFP control expression construct (ctrl; grey) are also shown. **c** FACS histogram of HCT116 cells showing membrane expression levels of TRAIL-R1 (red) and TRAIL-R2 (blue). The FACS profile of the isotype control is shown as filled black. **d** Supernatants from HEK293 cells transfected with either an EGFP control expression plasmid (grey), sTRAIL^wt^ (yellow), sTRAIL^DR5–1^ (dark green), sTRAIL^DR5–2^ (light green), sTRAIL^DR4–1^ (dark blue), sTRAIL^DR4–2^ (light blue) or sTRAIL^DR4–3^ (blue-grey) were normalised to 2 ng/ml TRAIL (the EGFP control was diluted 1:2 in fresh medium) and then applied to HCT116 (left), HCT.shDR4 (centre) and HCT.shDR5 cells (right), respectively, before apoptosis was measured 24 h later

We used soluble TRAIL (sTRAIL) expression constructs that we described previously [31, 36] to address the TRAIL-receptor preference in pancreatic cancer. These constructs contain an exogenous signal peptide sequence from the human fibrillin-1 gene, a cleavage site for the ubiquitous protease Furin, an Isoleucine Zipper domain and the ectodomain of TRAIL (aa114-aa281). In addition to the wild-type TRAIL construct (sTRAIL^wt^), we engineered constructs expressing three different DR4-specific sTRAIL variants (termed sTRAIL^DR4–1^, sTRAIL^DR4–2^ and sTRAIL^DR4–3^) and two specific for DR5 (labelled sTRAIL^DR5–1^ and sTRAIL^DR5–2^). These sTRAIL^DR4^ and sTRAIL^DR5^ variants contained various amino acid changes (Fig. 1a) [26, 44]. Following transfection of HEK293 cells we could demonstrate that all TRAIL variants were expressed and secreted to comparable levels (Fig. 1b). TRAIL receptor specificity was confirmed in HCT116 cells silenced for TRAIL-R1 and TRAIL-R2, respectively. We chose HCT116 cells, because of their relatively balanced TRAIL-receptor preference and expression levels (Fig. 1c). Cells with knocked-down TRAIL-R1 showed decreased apoptosis with all three sTRAIL^DR4^ variants, but elevated levels with sTRAIL^DR5^ as compared to sTRAIL^wt^ (Fig. 1d). In contrast, cells with silenced TRAIL-R2 exhibited markedly reduced apoptosis in response to sTRAIL^DR5^, whereas the levels of cell death were increased with the sTRAIL^DR4^ variants, in particular with sTRAIL^DR4–3^ (Fig. 1d). The likely reason for this observation is that in TRAIL-R1 and TRAIL-R2 silenced cells the chance of homotrimer formation is increased with sTRAIL variants when compared to sTRAIL^wt^ and parental cells resulting in higher apoptosis levels [24, 44].

### Induction of apoptosis by TRAIL variants in pancreatic cancer cells

**Fig. 2:**
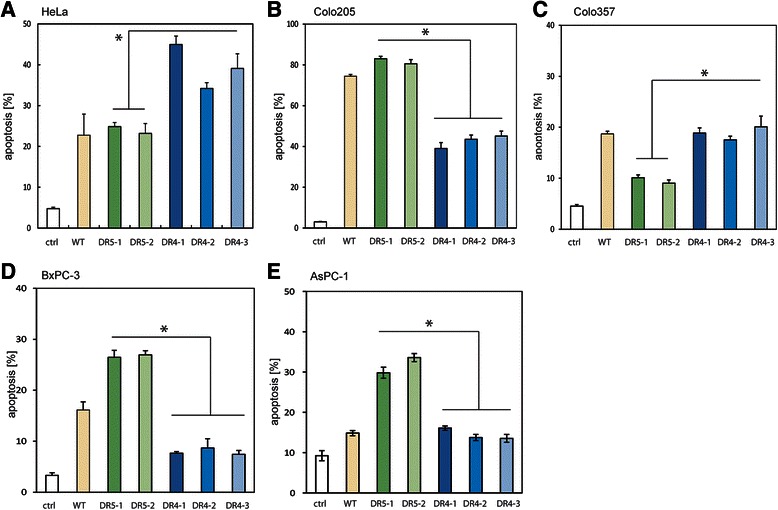
The TRAIL receptor preference for apoptosis induction is variable in pancreatic cancer cells. **a-e** Supernatants from HEK293 cells that were transfected with the EGFP control expression construct (ctrl; grey; 1:2 diluted), sTRAIL^wt^ (yellow; 2 ng/ml), sTRAIL^DR5–1^ (dark green; 2 ng/ml), sTRAIL^DR5–2^ (light green; 2 ng/ml), sTRAIL^DR4–1^ (dark blue; 2 ng/ml), sTRAIL^DR4–2^ (light blue; 2 ng/ml) or sTRAIL^DR4–3^ (blue-grey; 2 ng/ml) were then transferred onto (**a**) HeLa cells (prototypic DR4 specific cell type), (**b**) Colo205 cells (prototypic DR5 specific cell type), (**c**) Colo357 pancreatic cancer cells, (**d**) BxPC-3 pancreatic cancer cells and (**e**) AsPC-1 pancreatic cancer cells. After 24 h apoptosis was measured

Next, we tested several pancreatic cancer cells with the array of TRAIL variants. In parallel, we analysed cancer cells for which TRAIL-receptor preferences have been clearly documented, namely HeLa cells (TRAIL-R1) and Colo205 cells (TRAIL-R2). Next, we applied the sTRAIL variants to the pancreatic cancer cells Colo357, BxPC-3 and AsPC-1. After 24 h exposure to the sTRAIL variants we measured apoptosis and found that HeLa (Fig. 2a) and Colo205 (Fig. 2b) cells showed higher cell death levels with sTRAIL^DR4^ and sTRAIL^DR5^, respectively. However, while Colo357 pancreatic cancer cells exhibited elevated cell death rates with sTRAIL^DR4^ (Fig. 2c) as reported previously [45], BxPC-3 (Fig. 2d) and AsPC-1 (Fig. 2e) cells responded with higher apoptosis levels to sTRAIL^DR5^.

### TRAIL-receptor expression profile is not associated with receptor preferences

**Fig. 3:**
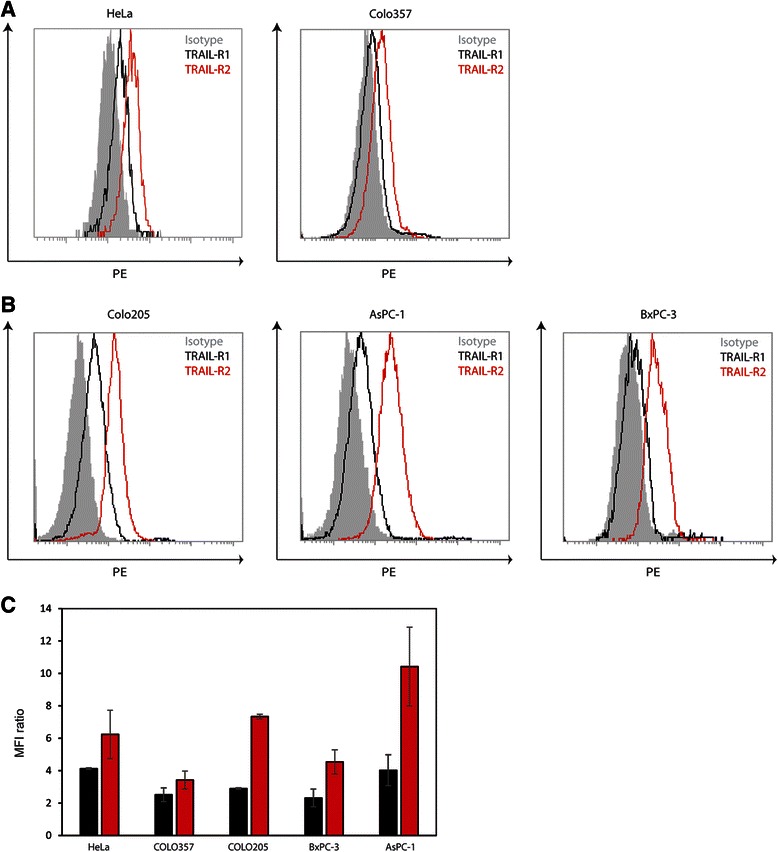
TRAIL-receptor surface expression profiles of pancreatic cancer cells and control cell lines. **a** FACS histograms of HeLa and Colo357 cells showing membrane expression levels of TRAIL-R1 (black) and TRAIL-R2 (red). The FACS profile of the isotype control is shown as filled grey. **b** FACS histograms of Colo205, AsPC-1 and BxPC-3 cells showing membrane expression levels of TRAIL-R1 (black) and TRAIL-R2 (red). The FACS profile of the isotype control is shown as filled grey. **c** Quantification of the FACS results for TRAIL-R1 (black) and TRAIL-R2 (red) for HeLa, Colo357, Colo205, AsPC-1 and BxPC-3 cells. The surface expression levels of the two receptors are expressed as MFI ratios

Next, we analysed whether the observed preferences for either TRAIL-R1 (HeLa, Colo357) or TRAIL-R2 (Colo205, BxPC-3, AsPC-1) could be linked to the surface expression levels of the two receptors. Using PE-conjugated antibodies against TRAIL-R1 and TRAIL-R2 and the appropriate isotype control, we found that HeLa cells harboured robust levels of TRAIL-R1 on their cell surface (MFI ratio: 4.12 +/− 0.05), whereas on Colo357 cells, we could detect only comparably low levels of TRAIL-R1 (MFI ratio: 2.51 +/− 0.43) (Fig. 3a). TRAIL-R2 levels in both HeLa and Colo357 cells are slightly higher than TRAIL-R1 (TRAIL-R2 MFI ratios: HeLa: 6.24 +/− 1.49 and Colo357: 3.42 +/− 0.55) (Fig. 3a). In the group of cells that preferentially responded to sTRAIL^DR5^ all cells showed higher levels of TRAIL-R2 (MFI ratios: Colo205: 7.33 +/− 0.14, AsPC-1: 10.42 +/− 2.43, BxPC-3: 4.54 +/− 0.75) than TRAIL-R1 (MFI ratios: Colo205: 2.90 +/− 0.04, AsPC-1: 4.02 +/− 0.96, BxPC-3: 2.31 +/− 0.55) (Fig. 3b), with all of them expressing levels that are not distinguishable from the group of cells reacting better to sTRAIL^DR4^. Thus, there is no straightforward correlation between the levels of TRAIL-R1 and TRAIL-R2 and TRAIL-receptor preference in TRAIL-induced apoptosis.

### Induction of apoptosis in sensitised TRAIL resistant pancreatic cancer cells

**Fig. 4:**
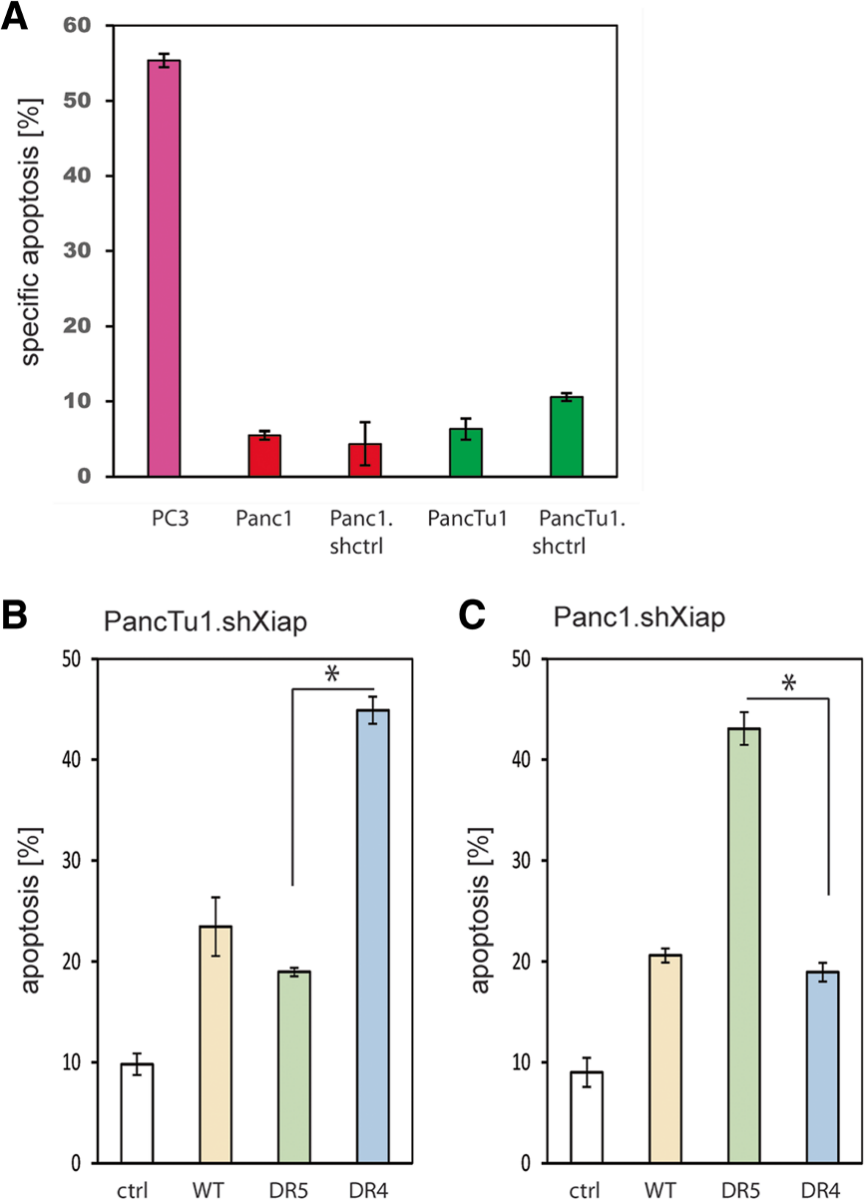
TRAIL receptor preference is also variable in apoptosis-sensitised pancreatic cancer cells. **a** TRAIL-sensitive control cells (PC-3), Panc1, Panc1.shctrl, PancTu1 and PancTu1.shctrl were treated with 10 ng/ml rTRAIL for 24 h, before apoptosis was measured. **b** PancTu1.shXIAP cells were treated with supernatants from HEK293 cells that were transfected with the EGFP control expression construct (ctrl; grey), sTRAIL^wt^ (yellow), sTRAIL^DR5–2^ (light green) or sTRAIL^DR4–3^ (blue-grey). After 24 h apoptosis was measured. **c** Panc1.shXIAP cells were treated with supernatants from HEK293 cells that were transfected with the EGFP control expression construct (ctrl; grey), sTRAIL^wt^ (yellow), sTRAIL^DR5–2^ (light green) or sTRAIL^DR4–3^ (blue-grey). After 24 h apoptosis was measured

It is well known that some pancreatic cancer cells are resistant to TRAIL (Fig. 4a). Therefore, in order to examine the TRAIL receptor preference in such cells, we silenced the anti-apoptotic protein XIAP in PancTu1 (PancTu1.shXIAP) and Panc1 (Panc1.shXIAP) cells and treated them with sTRAIL variants. The results show that knocking down of XIAP sensitised the cells to TRAIL-induced apoptosis, with sTRAIL^DR4^ having a significantly better effect in PancTu1.shXIAP (Fig. 4b), as previously described, but sTRAIL^DR5^ leading to more apoptosis in Panc1.shXIAP (Fig. 4c). Thus not all pancreatic cancer cells possess a preference for the TRAIL-R1 apoptosis pathway as reported previously [39, 40]. Instead, a group of pancreatic cancer cells have a higher propensity to undergo TRAIL-induced apoptosis via TRAIL-R2.

### TRAIL-receptor expression profile is not associated with receptor preferences in XIAP-silenced pancreatic cancer cells

**Fig. 5:**
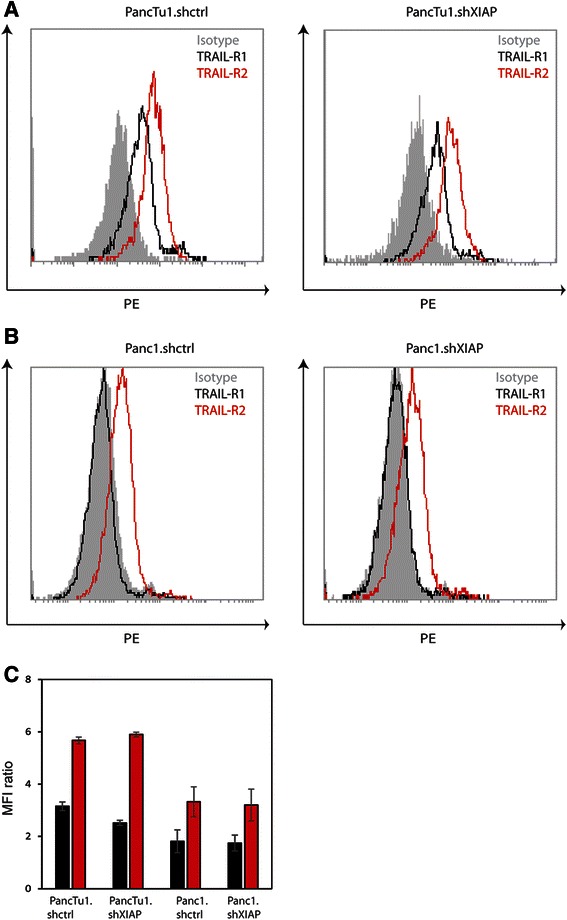
TRAIL-receptor surface expression profiles of TRAIL resistant pancreatic cancer cells and their XIAP silenced counterparts. **a** FACS histograms of Panc1.shctrl and Panc1.shXIAP cells showing membrane expression levels of TRAIL-R1 (black) and TRAIL-R2 (red). The FACS profile of the isotype control is shown as filled grey. **b** FACS histograms of PancTu1.shctrl and PancTu1.shXIAP cells showing membrane expression levels of TRAIL-R1 (black) and TRAIL-R2 (red). The FACS profile of the isotype control is shown as filled grey. **c** Quantification of the FACS results for TRAIL-R1 (black) and TRAIL-R2 (red) for Panc1.shctrl, Panc1.shXIAP, PancTu1.shctrl and PancTu1.shXIAP cells. The surface expression levels of the two receptors are expressed as MFI ratios

Next, we also measured TRAIL-R1 and TRAIL-R2 expression on the surface of both Panc1.shctrl and PancTu1.shctrl cells as well as their XIAP-silenced counterparts, Panc1.shXIAP and PancTu1.shXIAP cells. We found that the profiles of TRAIL-receptor expression did not differ between the control cells (Panc1.shctrl and PancTu1.shctrl) and the corresponding XIAP knock-down clones (Panc1.shXIAP and PancTu1.shXIAP) (Fig. 5a and b). TRAIL-R1 expression was almost undetectable in Panc1.shctrl and Panc1.shXIAP (MFI ratios: Panc1.shctrl: 1.81 +/− 0.44 and Panc1.shXIAP: 1.74 +/− 0.30), whereas TRAIL-R2 expression was readily detectable (MFI ratios: Panc1.shctrl: 3.33 +/− 0.57 and Panc1.shXIAP: 3.2 +/− 0.60). In PancTu1.shctrl and PancTu1.shXIAP both TRAIL-R1 and TRAIL-R2 were expressed at robust levels (TRAIL-R1 MFI ratios: PancTu1.shctrl: 3.15 +/− 0.17 and PancTu1.shXIAP: 2.52 +/− 0.10; TRAIL-R2 MFI ratios: PancTu1.shctrl: 5.67 +/− 0.13 and PancTu1.shXIAP: 5.90 +/− 0.08). This comparison of TRAIL-receptor levels in TRAIL resistant pancreatic cells also does not show a clear correlation between TRAIL-receptor expression levels and TRAIL-receptor preference after XIAP sensitisation.

## Discussion

Initially it was thought that TRAIL-R2 is the main apoptosis-inducing receptor for the death ligand TRAIL [27]. This led to the development and testing of agonistic antibodies against this receptor as potential anti-cancer agents [16, 18, 46, 47]. However, more recently reports showed that TRAIL-R1 has a more prominent role, than first thought, in specific types of cancer such as lymphoid malignancies [29] and leukaemic cells [30, 48]. Additionally, it was suggested that pancreatic cancer cells also trigger TRAIL-induced apoptosis mainly through TRAIL-R1 [39, 40]. However, when we analysed a wider array of pancreatic cancer cell lines we found that 2 out of 3 pancreatic cancer cells preferred the TRAIL-R2 pathway in response to TRAIL. In addition, Panc1 cells also showed higher apoptosis levels when treated with sTRAIL^DR5^ and XIAP was silenced concomitantly (Table 1).

**Table 1:** TRAIL-R preference of different cancer cell types

Cell line	Cancer cell type	TRAIL-receptor preference
HCT116	colorectal carcinoma	DR5
Colo205	colorectal carcinoma	DR5
HeLa	cervical carcinoma	DR4
Colo357	pancreatic carcinoma	DR4
BxPC-3	pancreatic carcinoma	DR5
AsPC-1	pancreatic carcinoma	DR5
Panc1	pancreatic carcinoma	DR5
PancTu1	pancreatic carcinoma	DR4

While these results appear to contrast the two afore mentioned publications [39, 40], it is important to point out that we used, at least in part, different cell lines and sTRAIL variant proteins instead of agonistic antibodies. Interestingly, the results in one of the reports indicate that both TRAIL-R1 and TRAIL-R2 agonistic antibodies can trigger apoptosis in pancreatic cells and that the TRAIL-R1 preference was only detected when one of the two receptors was inhibited by blocking antibodies followed by treatment with TRAIL [39]. In contrast, the second study found clear differences between the apoptosis-inducing activities of the two agonistic antibodies, with a clear preference for TRAIL-R1. It is therefore possible that sTRAIL variant proteins and TRAIL-receptor specific antibodies have distinct effects owing to their different modes of action with regard to their receptor engagement. Notwithstanding, the notion that pancreatic cancer cells and possibly other tumour types have a general TRAIL receptor preference needs to be re-visited, re-examined and possibly refined. Furthermore, we tested whether the expression profile of TRAIL-R1 and TRAIL-R2 could determine receptor preference, but failed to observe any clear correlation. These findings are generally in line with results reported earlier [39]. Thus, other factors and mechanisms than surface expression levels of the TRAIL-receptors must determine their apoptosis-inducing function.

Potential molecular mechanisms that could determine whether a receptor can be activated are O-glycosylation of both receptors [49] as well as S-palmitoylation, S-nitrosylation, N-glycosylation and ubiquitination of TRAIL-R1 [50–53]. Thus, despite being present on the cell surface a receptor might be relatively inactive, making it impossible to determine receptor preferences based solely on expression levels.

An area where specific TRAIL variants and/or agonistic antibodies can be used with good predictability is in combination treatments, in which up-regulation of either TRAIL-R1 or TRAIL-R2 can be targeted by the respective variant. For example, pre-treatment with the anti-cancer drug doxorubicin gave rise to significantly increased cell death when treated with the agonistic TRAIL-R2 antibody lexatumumab [54]. In addition, combined treatment of colorectal tumours with lexatumumab and radiotherapy had similar sensitising effects [55]. Soluble TRAIL^DR5^ also showed better apoptosis inducing effects after priming with 5-Fluorouracil as compared to sTRAIL^wt^ or sTRAIL^DR4^, because the drug caused p53-independent upregulation of TRAIL-R2 [31]. In contrast, HDAC inhibition has been shown to result in sensitisation to TRAIL-R1 specific apoptosis [48, 56]. Of note in this context is that the individual activation of TRAIL-R1 and -R2 could be an advantage, since it was shown that combined exposure to DR4- and DR5-selective TRAIL variants in cells, sensitive for both receptors, was more potent in triggering apoptosis when compared to single agent treatment [22]. Other factors that can influence TRAIL receptor preferences are so called non-canonical pathways including the activation of NF-κB, p38 and JNK [57]. The issue with these pathways is that they have been reported to have opposing effects and different apoptosis factor requirements depending on cell type and cellular context [57]. For example, TRAIL-induced JNK activation has been reported to be caspase-dependent in HeLa human cervical cancer cells, but caspase-independent in the human rhabdomyosarcoma Kym-1 cell line [58]. These findings illustrate that the TRAIL receptors have varying, cell type-specific and in parts receptor specific capabilities to recruit different signalling complexes to their intracellular domain. These complexes and their individual constituents might have an impact on the apoptosis-inducing function of the receptors and thereby may contribute to TRAIL-receptor preferences in TRAIL-triggered cell death.

Consequently, further research is needed to better understand potential differences between TRAIL agonistic antibodies and recombinant TRAIL proteins and variants. Additionally, it is important to elucidate the molecular components that determine TRAIL-receptor preferences in order to be able to select the best TRAIL agents to potentially treat pancreatic cancer and other tumour types in the future.

## Conclusions

We discovered that not all pancreatic cancer cells favour the TRAIL-R1 pathway to induce apoptosis and that no clear and direct correlation exists between the surface expression levels of TRAIL-R1 and TRAIL-R2 and their preference for one of the two receptors. AsPC-1, BxPC-3 and Panc1 cells elicit apoptosis via TRAIL-R2, whereas Colo357 cells and PancTu1 cells preferred TRAIL-R1 to induce cell death. Thus, claims of general cancer type specific TRAIL receptor preference should be taken with a pinch of salt.

## Abbreviations

ANOVA: Analysis of variance between groups
CMV: Cytomegalie virus
DISC: Death-inducing signaling complex
DMEM: Dulbecco’s modified Eagle’s medium
EGFP: Enhanced green fluorescent protein
ELISA: Enzyme-linked Immunosorbent Assay
FACS: Fluorescence-activated Cell Sorting
FADD: Fas-associated death domain
FBS: Fetal bovine serum
FIB: Fibrillin
Furin CS: Furin cleavage site
HBS: Hepes-buffered saline
ILZ: Isoleucine zipper
JNK: c-Jun N-terminal kinase
NF-κB: Nuclear factor kappa-light-chain-enhancer of activated B cells
OPG: Osteoprogerin
PBS: Phosphate-buffered saline
PE: R-Phycoerythrin
RNAi: RNA interference
RPMI 1640 medium: Roswell Park Memorial Institute 1640 medium
SEM: Standard error of the mean
TRAIL: TNF-related apoptosis-inducing ligand
sTRAIL: soluble TNF-related apoptosis-inducing ligand
TRAIL-R1/DR4: TRAIL-receptor 1/Death-receptor 4
TRAIL-R2/DR5: TRAIL-receptor 2/ Death-receptor 5
TRAIL-R3/DcR1: TRAIL-receptor 3/Decoy-receptor 1
TRAIL-R4/DcR2: TRAIL-receptor 4/Decoy-receptor 2
XIAP: X-linked Inhibitor of apoptosis protein

## References

[1] Duiker EW, Mom CH, de Jong S, Willemse PH, Gietema JA, van der Zee AG (2006). The clinical trail of TRAIL. Eur J Cancer.

[2] Lemke J, von Karstedt S, Zinngrebe J, Walczak H (2014). Getting TRAIL back on track for cancer therapy. Cell Death Differ.

[3] Wiley SR, Schooley K, Smolak PJ, Din WS, Huang CP, Nicholl JK (1995). Identification and characterization of a new member of the TNF family that induces apoptosis. Immunity.

[4] Wu GS (2009). TRAIL as a target in anti-cancer therapy. Cancer Lett.

[5] Micheau O, Shirley S, Dufour F (2013). Death receptors as targets in cancer. Br J Pharmacol.

[6] Chaudhary PM, Eby M, Jasmin A, Bookwalter A, Murray J, Hood L (1997). Death receptor 5, a new member of the TNFR family, and DR4 induce FADD- dependent apoptosis and activate the NF-kappaB pathway. Immunity.

[7] Schneider P, Thome M, Burns K, Bodmer JL, Hofmann K, Kataoka T (1997). TRAIL receptors 1 (DR4) and 2 (DR5) signal FADD-dependent apoptosis and activate NF-kappaB. Immunity.

[8] Mahalingam D, Szegezdi E, Keane M, de Jong S, Samali A (2009). TRAIL receptor signalling and modulation: Are we on the right TRAIL?. Cancer Treat Rev.

[9] Sprick MR, Weigand MA, Rieser E, Rauch CT, Juo P, Blenis J (2000). FADD/MORT1 and caspase-8 are recruited to TRAIL receptors 1 and 2 and are essential for apoptosis mediated by TRAIL receptor 2. Immunity.

[10] Hellwig CT, Rehm M (2012). TRAIL signaling and synergy mechanisms used in TRAIL-based combination therapies. Mol Cancer Ther.

[11] Bratton SB, MacFarlane M, Cain K, Cohen GM (2000). Protein complexes activate distinct caspase cascades in death receptor and stress-induced apoptosis. Exp Cell Res.

[12] Degli-Esposti MA, Dougall WC, Smolak PJ, Waugh JY, Smith CA, Goodwin RG (1997). The novel receptor TRAIL-R4 induces NF-kappaB and protects against TRAIL-mediated apoptosis, yet retains an incomplete death domain. Immunity.

[13] Degli-Esposti MA, Smolak PJ, Walczak H, Waugh J, Huang CP, DuBose RF (1997). Cloning and Characterization of TRAIL-R3, a Novel Member of the Emerging TRAIL Receptor Family. J Exp Med.

[14] Emery JG, McDonnell P, Burke MB, Deen KC, Lyn S, Silverman C (1998). Osteoprotegerin is a receptor for the cytotoxic ligand TRAIL. J Biol Chem.

[15] LeBlanc HN, Ashkenazi A (2003). Apo2L/TRAIL and its death and decoy receptors. Cell Death Differ.

[16] Camidge DR, Herbst RS, Gordon MS, Eckhardt SG, Kurzrock R, Durbin B (2010). A phase I safety and pharmacokinetic study of the death receptor 5 agonistic antibody PRO95780 in patients with advanced malignancies. Clin Cancer Res.

[17] Chuntharapai A, Dodge K, Grimmer K, Schroeder K, Marsters SA, Koeppen H (2001). Isotype-dependent inhibition of tumor growth in vivo by monoclonal antibodies to death receptor 4. J Immunol.

[18] Ichikawa K, Liu W, Zhao L, Wang Z, Liu D, Ohtsuka T (2001). Tumoricidal activity of a novel anti-human DR5 monoclonal antibody without hepatocyte cytotoxicity. Nat Med.

[19] Trarbach T, Moehler M, Heinemann V, Kohne CH, Przyborek M, Schulz C (2010). Phase II trial of mapatumumab, a fully human agonistic monoclonal antibody that targets and activates the tumour necrosis factor apoptosis-inducing ligand receptor-1 (TRAIL-R1), in patients with refractory colorectal cancer. Br J Cancer.

[20] van Geelen CM, Pennarun B, Le PT, de Vries EG, de Jong S (2011). Modulation of TRAIL resistance in colon carcinoma cells: different contributions of DR4 and DR5. BMC Cancer..

[21] den Hollander MW, Gietema JA, de Jong S, Walenkamp AM, Reyners AK, Oldenhuis CN (2013). Translating TRAIL-receptor targeting agents to the clinic. Cancer Lett.

[22] Reis CR, van der Sloot AM, Natoni A, Szegezdi E, Setroikromo R, Meijer M (2010). Rapid and efficient cancer cell killing mediated by high-affinity death receptor homotrimerizing TRAIL variants. Cell Death Dis..

[23] Reis CR, van der Sloot AM, Szegezdi E, Natoni A, Tur V, Cool RH (2009). Enhancement of antitumor properties of rhTRAIL by affinity increase toward its death receptors. Biochemistry (Mosc).

[24] Szegezdi E, van der Sloot AM, Mahalingam D, O’Leary L, Cool RH, Munoz IG (2012). Kinetics in signal transduction pathways involving promiscuous oligomerizing receptors can be determined by receptor specificity: apoptosis induction by TRAIL. Mol Cell Proteomics.

[25] Tur V, van der Sloot AM, Reis CR, Szegezdi E, Cool RH, Samali A (2008). DR4-selective tumor necrosis factor-related apoptosis-inducing ligand (TRAIL) variants obtained by structure-based design. J Biol Chem.

[26] van der Sloot AM, Tur V, Szegezdi E, Mullally MM, Cool RH, Samali A (2006). Designed tumor necrosis factor-related apoptosis-inducing ligand variants initiating apoptosis exclusively via the DR5 receptor. Proc Natl Acad Sci U S A.

[27] Kelley RF, Totpal K, Lindstrom SH, Mathieu M, Billeci K, DeForge L (2005). Receptor-selective mutants of apoptosis-inducing ligand 2/tumor necrosis factor-related apoptosis-inducing ligand reveal a greater contribution of death receptor (DR) 5 than DR4 to apoptosis signaling. J Biol Chem.

[28] Duiker EW, de Vries EG, Mahalingam D, Meersma GJ, Boersma-van Ek W, Hollema H (2009). Enhanced antitumor efficacy of a DR5-specific TRAIL variant over recombinant human TRAIL in a bioluminescent ovarian cancer xenograft model. Clin Cancer Res.

[29] MacFarlane M, Kohlhaas SL, Sutcliffe MJ, Dyer MJ, Cohen GM (2005). TRAIL receptor-selective mutants signal to apoptosis via TRAIL-R1 in primary lymphoid malignancies. Cancer Res.

[30] Szegezdi E, Reis CR, van der Sloot AM, Natoni A, O’Reilly A, Reeve J (2011). Targeting AML through DR4 with a novel variant of rhTRAIL. J Cell Mol Med.

[31] Yu R, Deedigan L, Albarenque SM, Mohr A, Zwacka RM (2013). Delivery of sTRAIL variants by MSCs in combination with cytotoxic drug treatment leads to p53-independent enhanced antitumor effects. Cell Death Dis..

[32] Meijer A, Kruyt FA, van der Zee AG, Hollema H, Le P, ten Hoor KA (2013). Nutlin-3 preferentially sensitises wild-type p53-expressing cancer cells to DR5-selective TRAIL over rhTRAIL. Br J Cancer.

[33] Kim CY, Jeong M, Mushiake H, Kim BM, Kim WB, Ko JP (2006). Cancer gene therapy using a novel secretable trimeric TRAIL. Gene Ther.

[34] Kim SM, Lim JY, Park SI, Jeong CH, Oh JH, Jeong M (2008). Gene therapy using TRAIL-secreting human umbilical cord blood-derived mesenchymal stem cells against intracranial glioma. Cancer Res.

[35] Menon LG, Kelly K, Yang HW, Kim SK, Black PM, Carroll RS (2009). Human bone marrow-derived mesenchymal stromal cells expressing S-TRAIL as a cellular delivery vehicle for human glioma therapy. Stem Cells.

[36] Mohr A, Albarenque SM, Deedigan L, Yu R, Reidy M, Fulda S (2010). Targeting of XIAP Combined with Systemic Mesenchymal Stem Cell-Mediated Delivery of sTRAIL Ligand Inhibits Metastatic Growth of Pancreatic Carcinoma Cells. Stem Cells.

[37] Mohr A, Henderson G, Dudus L, Herr I, Kuerschner T, Debatin KM (2004). AAV-encoded expression of TRAIL in experimental human colorectal cancer leads to tumor regression. Gene Ther.

[38] Mohr A, Lyons M, Deedigan L, Harte T, Shaw G, Howard L (2008). Mesenchymal stem cells expressing TRAIL lead to tumour growth inhibition in an experimental lung cancer model. J Cell Mol Med.

[39] Lemke J, Noack A, Adam D, Tchikov V, Bertsch U, Roder C (2010). TRAIL signaling is mediated by DR4 in pancreatic tumor cells despite the expression of functional DR5. J Mol Med.

[40] Stadel D, Mohr A, Ref C, MacFarlane M, Zhou S, Humphreys R (2010). TRAIL-induced apoptosis is preferentially mediated via TRAIL receptor 1 in pancreatic carcinoma cells and profoundly enhanced by XIAP inhibitors. Clin Cancer Res.

[41] Buneker CK, Yu R, Deedigan L, Mohr A, Zwacka RM (2012). IFN-gamma combined with targeting of XIAP leads to increased apoptosis-sensitisation of TRAIL resistant pancreatic carcinoma cells. Cancer Lett.

[42] Mohr A, Buneker C, Gough RP, Zwacka RM (2008). MnSOD protects colorectal cancer cells from TRAIL-induced apoptosis by inhibition of Smac/DIABLO release. Oncogene.

[43] Nicoletti I, Migliorati G, Pagliacci MC, Grignani F, Riccardi C (1991). A rapid and simple method for measuring thymocyte apoptosis by propidium iodide staining and flow cytometry. J Immunol Methods.

[44] Reis CR, van Assen AH, Quax WJ, Cool RH (2011). Unraveling the binding mechanism of trivalent tumor necrosis factor ligands and their receptors. Mol Cell Proteomics.

[45] Yu R, Albarenque SM, Cool RH, Quax WJ, Mohr A, Zwacka RM (2014). DR4 specific TRAIL variants are more efficacious than wild-type TRAIL in pancreatic cancer. Cancer Biol Ther.

[46] Buchsbaum DJ, Zhou T, Grizzle WE, Oliver PG, Hammond CJ, Zhang S (2003). Antitumor efficacy of TRA-8 anti-DR5 monoclonal antibody alone or in combination with chemotherapy and/or radiation therapy in a human breast cancer model. Clin Cancer Res.

[47] Muhlenbeck F, Schneider P, Bodmer JL, Schwenzer R, Hauser A, Schubert G (2000). The tumor necrosis factor-related apoptosis-inducing ligand receptors TRAIL-R1 and TRAIL-R2 have distinct cross-linking requirements for initiation of apoptosis and are non-redundant in JNK activation. J Biol Chem.

[48] MacFarlane M, Inoue S, Kohlhaas SL, Majid A, Harper N, Kennedy DB (2005). Chronic lymphocytic leukemic cells exhibit apoptotic signaling via TRAIL-R1. Cell Death Differ.

[49] Wagner KW, Punnoose EA, Januario T, Lawrence DA, Pitti RM, Lancaster K (2007). Death-receptor O-glycosylation controls tumor-cell sensitivity to the proapoptotic ligand Apo2L/TRAIL. Nat Med.

[50] Rossin A, Derouet M, Abdel-Sater F, Hueber AO (2009). Palmitoylation of the TRAIL receptor DR4 confers an efficient TRAIL-induced cell death signalling. Biochem J.

[51] Tang Z, Bauer JA, Morrison B, Lindner DJ (2006). Nitrosylcobalamin promotes cell death via S nitrosylation of Apo2L/TRAIL receptor DR4. Mol Cell Biol.

[52] Yoshida T, Shiraishi T, Horinaka M, Wakada M, Sakai T (2007). Glycosylation modulates TRAIL-R1/death receptor 4 protein: different regulations of two pro-apoptotic receptors for TRAIL by tunicamycin. Oncol Rep.

[53] van de Kooij B, Verbrugge I, de Vries E, Gijsen M, Montserrat V, Maas C (2013). Ubiquitination by the membrane-associated RING-CH-8 (MARCH-8) ligase controls steady-state cell surface expression of tumor necrosis factor-related apoptosis inducing ligand (TRAIL) receptor 1. J Biol Chem.

[54] Wu XX, Jin XH, Zeng Y, El Hamed AM, Kakehi Y (2007). Low concentrations of doxorubicin sensitizes human solid cancer cells to tumor necrosis factor-related apoptosis-inducing ligand (TRAIL)-receptor (R) 2-mediated apoptosis by inducing TRAIL-R2 expression. Cancer Sci.

[55] Marini P, Denzinger S, Schiller D, Kauder S, Welz S, Humphreys R (2006). Combined treatment of colorectal tumours with agonistic TRAIL receptor antibodies HGS-ETR1 and HGS-ETR2 and radiotherapy: enhanced effects in vitro and dose-dependent growth delay in vivo. Oncogene.

[56] Natoni A, MacFarlane M, Inoue S, Walewska R, Majid A, Knee D (2007). TRAIL signals to apoptosis in chronic lymphocytic leukaemia cells primarily through TRAIL-R1 whereas cross-linked agonistic TRAIL-R2 antibodies facilitate signalling via TRAIL-R2. Br J Haematol.

[57] Azijli K, Weyhenmeyer B, Peters GJ, de Jong S, Kruyt FA (2013). Non-canonical kinase signaling by the death ligand TRAIL in cancer cells: discord in the death receptor family. Cell Death Differ.

[58] Muhlenbeck F, Haas E, Schwenzer R, Schubert G, Grell M, Smith C (1998). TRAIL/Apo2L activates c-Jun NH2-terminal kinase (JNK) via caspase-dependent and caspase-independent pathways. J Biol Chem.

